# Supplementary material for: Parallel ventral hippocampus-lateral septum pathways differentially regulate approach-avoidance conflict
Source: Nat Commun. 2022 Jun 10;13:3349. doi: 10.1038/s41467-022-31082-0 (PMC9187740; doi:10.1038/s41467-022-31082-0)
Supplement: Supplementary file 1 — Supplementary Information [file 41467_2022_31082_MOESM1_ESM.docx]

**Supplementary Information**

**Supplementary Figure 1: Mixed valence approach-avoidance conflict task acquisition test results. a,e** All groups spent the most time in the appetitively cued arm and the least time in the aversively cued arm (vCA3 Arm: F_(2,38)_ = 118.57, P = 0.0001; vCA1 Arm: F_(2,36)_ = 59.09, P = 0.0001). **b,f** All groups entered the appetitive arm more than the other arms, and made the fewest entries into the aversive arm (vCA3 Arm: F_(2,38)_ = 53.56, P = 0.0001; vCA1 Arm: F_(2,36)_ = 35.25, P = 0.0001). **c,g** All groups stayed in the appetitive arm, but not the aversive or neutral arms (vCA3 Arm: F_(2,38)_ = 18.27, P < 0.0001; vCA1 Arm: F_(2,36)_ = 8.36, P = 0.0007). **d,h** All groups exhibited more retreats from the aversive arm than the other arms (vCA3 Arm: F_(2,38)_ = 16.14, P = 0.0003; vCA1 Arm: F_(2,36)_ = 7.58, P = 0.0018). vCA3-hM4Di n = 11, vCA3-GFP n = 10, vCA1-hM4Di n = 12, vCA1-GFP n = 8. All tests are two-way ANOVAs. Data represent mean ± SEM. Source data are provided as a Source Data file.

**
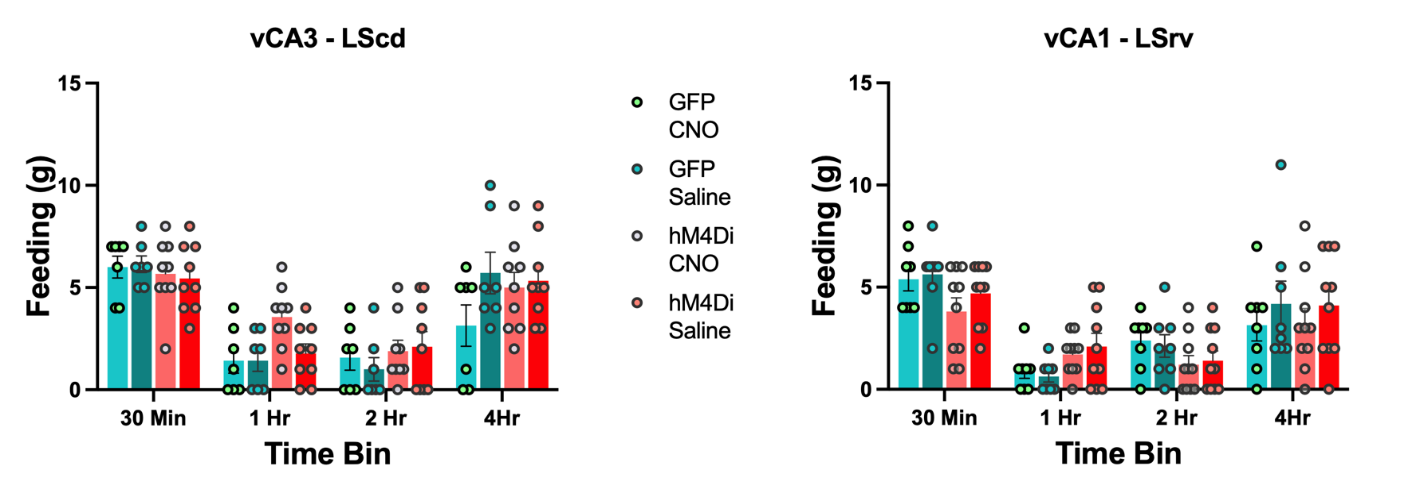
**

**Supplementary Figure 2: Bin by Bin Free Feeding Test Results.** Animals’ food consumption varied depending on the time bin in both vCA3 🡪 LScd groups (Time Bin: F_(3,42)_ = 30.35, P = 0.0001) and vCA3 🡪 LScd groups (Time Bin: F_(3,48)_ = 28.06, P = 0.0001). The vCA3 🡪 LScd groups showed a significant drug x bin interaction (F_(3,42)_ = 4.12, P = 0.0128), but no post hoc comparisons reached significance (Lowest P = 0.3676). The vCA1 🡪 LSrv groups also showed a significant virus x bin interaction (F_(3,48)_ = 2.99, P = 0.0386), with significantly lower food consumption in the 1 Hr time bin by the EGFP animals in both drug conditions (*t*_30.59_= -2.82, P = 0.0340). vCA3-hM4Di n = 9, vCA3-GFP n = 7, vCA1-hM4Di n = 10, vCA1-GFP n = 8. Tests are three-way repeated measures ANOVAs with drug and bin as within-subject factors and virus as a between-subject factor. Data represent mean ± SEM. Source data are provided as a Source Data file.


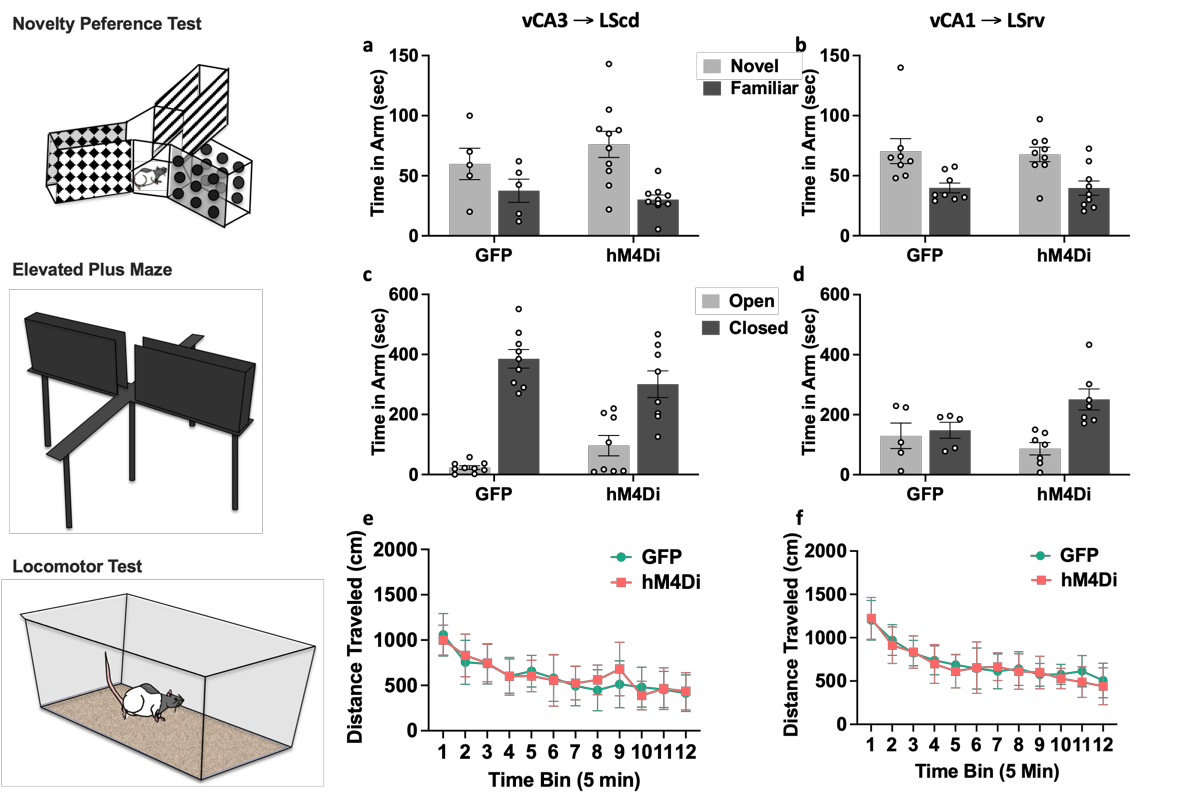


**Supplementary Figure 3. vHPC 🡪 LS circuit inhibition does not affect novelty detection, elevated plus maze behavior, or locomotor activity. a-b** Novelty detection was similar within vCA3 🡪 LScd groups (Arm: F_(1,12)_ = 12.45, P = 0.0039, Virus: F_(1,12)_ = 0.10, P = 0.7597; Virus x Arm: F_(1,12)_ = 1.42, P = 0.2542) and within vCA1 🡪 LSrv groups (Arm: F_(1,16)_ = 18.03, P = 0.0002, Virus: F_(1,16)_ = 0.18, P = 0.6940; Virus x Arm: F_(1,16)_ = 0.08, P = 0.7936). **c-d** EPM closed arm exploration was higher than open arm exploration, and was similar within vCA3 🡪 LScd groups (Arm: F_(1,15)_ = 49.67, P = 0.0001, Virus: F(_1,15_) = 0.10, P = 0.1003; Virus x Arm: F(1,15) = 3.85, P = 0.0726) and within vCA1 🡪 LSrv groups (Arm: F_(1,10)_ = 5.13, P = 0.0486, Virus: F_(1,10)_ = 2.24, P = 0.1669; Virus x Arm: F(_1,10_) = 3.26, P = 0.1031). **e-f** Locomotor activity declined overtime similarly for both vCA3 🡪 LScd (Time Bin: F_(11,220)_ = 25.77, P = 0.0001, Virus: F(_1,20_) = 0.07, P = 0.8101; Virus x Time Bin: F(_11,220_) = 1.16, P = 0.3219) and vCA1 🡪 LSrv groups (Time Bin: F_(11,220)_ = 38.32, P = 0.0001, Virus: F_(1,20)_ = 0.23, P = 0.6301; Virus x Time Bin: F_(1,220)_ = 0.63, P = 0.7976). In novelty detection test vCA3-hM4Di n = 9, vCA3-GFP n = 5, vCA1-hM4Di n = 10, vCA1-GFP n = 8. In EPM vCA3-hM4Di n = 8, vCA3-GFP n = 9, vCA1-hM4Di n = 7, vCA1-GFP n = 5. In locomotor test vCA3-hM4Di n = 12, vCA3-GFP n = 10, vCA1-hM4Di n = 13, vCA1-GFP n = 9. All tests are two-way ANOVAs. Data represent mean ± SEM. Source data are provided as a Source Data file.

**Supplementary Figure 4. Differential LScd and LSrv output tracing a** Schematic of GFP transduction of LScd. **b** Somatic GFP expression in LScd. **c-h** Anterograde projections of LScd. **i** Schematic of GFP transduction of LSrv. **j** Somatic GFP expression in LSrv. **k-p** Anterograde projections of LSrv. 0.5 mm scale bar for images **b-h** and **j-p**. 3V: 3^rd^ ventricle, ac: anterior commissure, AH: anterior hypothalamus, AHA: anterior hypothalamic area, BNST: bed nucleus of the stria terminalis, DMD: Dorsomedial hypothalamus dorsal part, DMH: Dorsomedial hypothalamus, ESO: episupraoptic nucleus, f: fornix, HDB: horizontal limb of the diagonal band, LA: lateroanterior hypothalamic nucleus, LPO: lateral preoptic area, LScd: caudodorsal lateral septum, LSrv: rostroventral lateral septum LV: lateral ventricle, MM: medial mammillary nucleus, ML: lateral mammillary nucleus, MPA: medial preoptic area, MPO: medial preoptic nucleus, MS: medial septum, MTu: medial tuberal nucleus, opt: optic tract, PaV: paraventricular nucleus, Pe: periventricular nucleus, PeFLH: perifornical part of the lateral hypothalamus, PLH: peduncular lateral hypothalamus, RCh: retrochiasmatic area, SHy: septohypothalamuic nucleus, SN: substantia nigra, SUM-Med: supramammillary nucleus medial, SUM-Lat: supramammillary nucleus lateral, Tu: olfactory tubercle, TuLH: tuberal region of the lateral hypothalamus, VLH: Ventrolateral hypothalamus, VMH: ventromedial hypothalamus, VMHSh: shell of the ventromedial hypothalamus, VTA: ventral tegmental area, ZI: Zona Incerta. Tracing experiments were conducted in 6 animals, which similar tracing patterns in each animal.
