# Supplementary material for: Parallel ventral hippocampus-lateral septum pathways differentially regulate approach-avoidance conflict
Source: Nat Commun. 2022 Jun 10;13:3349. doi: 10.1038/s41467-022-31082-0 (PMC9187740; doi:10.1038/s41467-022-31082-0)
Supplement: Supplementary file 3 — Reporting Summary [file 41467_2022_31082_MOESM3_ESM.pdf]

nature portfolio

Corresponding author(s): Rutsuko Ito

Last updated by author(s): Feb 13, 2022

Reporting Summary

Nature Portfolio wishes to improve the reproducibility of the work that we publish. This form provides structure for consistency and transparency in reporting. For further information on Nature Portfolio policies, see our Editorial Policies and the Editorial Policy Checklist.

Statistics

For all statistical analyses, confirm that the following items are present in the figure legend, table legend, main text, or Methods section.

n/a	Confirmed	
☐	☒	The exact sample size (*n*) for each experimental group/condition, given as a discrete number and unit of measurement
☐	☒	A statement on whether measurements were taken from distinct samples or whether the same sample was measured repeatedly
☐	☒	The statistical test(s) used AND whether they are one- or two-sided<br>*Only common tests should be described solely by name; describe more complex techniques in the Methods section.*
☒	☐	A description of all covariates tested
☐	☒	A description of any assumptions or corrections, such as tests of normality and adjustment for multiple comparisons
☐	☒	A full description of the statistical parameters including central tendency (e.g. means) or other basic estimates (e.g. regression coefficient) AND variation (e.g. standard deviation) or associated estimates of uncertainty (e.g. confidence intervals)
☐	☒	For null hypothesis testing, the test statistic (e.g. F, t, r) with confidence intervals, effect sizes, degrees of freedom and P value noted<br>*Give P values as exact values whenever suitable.*
☒	☐	For Bayesian analysis, information on the choice of priors and Markov chain Monte Carlo settings
☒	☐	For hierarchical and complex designs, identification of the appropriate level for tests and full reporting of outcomes
☒	☐	Estimates of effect sizes (e.g. Cohen's *d*, Pearson's *r*), indicating how they were calculated

Our web collection on statistics for biologists contains articles on many of the points above.

Software and code

Policy information about availability of computer code

Data collection	All behavioral data collection was conducted using manually operated apparatuses and hence no codes were used. Noldus EthoVision tracking software XT15 was used to record behaviour for subsequent scoring. Nikon NIS elements was used for brain image capture and processing. ImageJ2 v2.3.0/1.53f (Fiji) was used for cfos-positive cell quantification
Data analysis	All data were analyzed using R version 4.0.5 using the “tidyverse”(1.3.1), “MKinfer”(0.6), and “permuco” (1.1.1) packages, and graphed in GraphPad Prism version 8.1.1.

For manuscripts utilizing custom algorithms or software that are central to the research but not yet described in published literature, software must be made available to editors and reviewers. We strongly encourage code deposition in a community repository (e.g. GitHub). See the Nature Portfolio guidelines for submitting code & software for further information.

Data

Policy information about availability of data

All manuscripts must include a data availability statement. This statement should provide the following information, where applicable:

- Accession codes, unique identifiers, or web links for publicly available datasets
- A description of any restrictions on data availability
- For clinical datasets or third party data, please ensure that the statement adheres to our policy

All raw data are available are available in the supplementary source data file.

Field-specific reporting

Please select the one below that is the best fit for your research. If you are not sure, read the appropriate sections before making your selection.

☒ Life sciences ☐ Behavioural & social sciences ☐ Ecological, evolutionary & environmental sciences

For a reference copy of the document with all sections, see nature.com/documents/nr-reporting-summary-flat.pdf

Life sciences study design

All studies must disclose on these points even when the disclosure is negative.

Sample size	Sample size calculations were not conducted a priori. Instead, they were based upon previous publications from our laboratory (Schumacher et al., 2018; Ref10, Yeates et al., 2019, Ref11).
Data exclusions	Data exclusions were predetermined, with the exception of cases involving illness (n=4) and technical error during data collection (n=3 for NESF/FESF tests, n=6 for immunohistochemistry, 2 for preference/avoidance tests). Pre-estbalished exclusions occurred based on animals failing to learn the Y-maze task (n=12), misplacement of cannulae or viral expression (n=4), or animals failing to initiate contact with the food in the 10min NESF test (n=1).
Replication	We have replicated the same pattern of results in all reported experiments across 6 cohorts of animals (n=8-16) that were tested successively (n=3-4 in each group: vCA3-LScd hM4Di vs. vCA3-LScd EGFP; vCA1-LSrv hM4Di vs vCA1-LSrv EGFP), and are therefore confident of the reproducibility of our findings.
Randomization	Animals were randomly assigned to virus group (hM4Di, EGFP) and pathway (vCA3-LScd, vCA1-LSrv), and to drug group (CNO, Saline) for cfos analysis.
Blinding	The experimenters were not blinded to the group allocation of the animals while performing the experiment, primarily due to the fact that animals were tested in small batches of ~8. However, all behavioral data were video recorded and scored blinded to virus assignment and pathway group. Similarly, the cfos quantification was conducted blinded to the drug (CNO, saline).

Reporting for specific materials, systems and methods

We require information from authors about some types of materials, experimental systems and methods used in many studies. Here, indicate whether each material, system or method listed is relevant to your study. If you are not sure if a list item applies to your research, read the appropriate section before selecting a response.

Materials & experimental systems

n/a	Involved in the study	
☐	☒	Antibodies
☒	☐	Eukaryotic cell lines
☒	☐	Palaeontology and archaeology
☐	☒	Animals and other organisms
☒	☐	Human research participants
☒	☐	Clinical data
☒	☐	Dual use research of concern

Methods

n/a	Involved in the study	
☒	☐	ChIP-seq
☒	☐	Flow cytometry
☒	☐	MRI-based neuroimaging

Antibodies

Antibodies used	Rabbit anti-c-fos Synatpic Systems #226-003<br>Peroxidase AffiniPure Donkey Anti-Rabbit IgG (H+L) Jackson ImmunoResearch labs #711-035-152
Validation	The specificity of the primary anti-cfos antibody was validated in publications listed on CiteAb (e.g., Plaisier et al., 2020, J Neuroendocrinology, Hume & Menzies 2017, Endocrinology).

Animals and other organisms

Policy information about studies involving animals; ARRIVE guidelines recommended for reporting animal research

Laboratory animals	Male Long Evans rats from Charles River (6-8 weeks) were used for this study.
Wild animals	The study did not involve wild animals.
Field-collected samples	The study did not use field-collected samples.

Ethics oversight	All experiments were conducted in accordance with the guidelines of Canadian Council of Animal Care and approved by the Local Animal Care Committee at the University of Toronto.

Note that full information on the approval of the study protocol must also be provided in the manuscript.
